# Study on the Dynamic Biological Characteristics of Sca-1^+^ Hematopoietic Stem and Progenitor Cell Senescence

**DOI:** 10.1155/2015/954120

**Published:** 2015-05-27

**Authors:** Shan Geng, Xin-Yi Mu, Xiong-Bin Chen, Ji-Ying Hou, Dao-Yong Jia, Chun-Yan Xu, Ya-Ping Wang

**Affiliations:** ^1^Laboratory of Stem Cells and Tissue Engineering, Department of Histology and Embryology, Chongqing Medical University, Chongqing 400016, China; ^2^Chongqing Medical University, Chongqing 400016, China

## Abstract

The researches in the dynamic changes of the progress of HSCs aging are very limited and necessary. In this study, male C57BL/6 mice were divided into 5 groups by age. We found that the superoxide damage of HSPCs started to increase from the middle age (6 months old), with notably reduced antioxidation ability. In accordance with that, the senescence of HSPCs also started from the middle age, since the self-renewal and differentiation ability remarkably decreased, and senescence-associated markers SA-*β*-GAL increased in the 6-month-old and the older groups. Interestingly, the telomere length and telomerase activity increased to a certain degree in the 6-month-old group. It suggested an intrinsic spontaneous ability of HSPCs against aging. It may provide a theoretical and experimental foundation for better understanding the senescence progress of HSPCs. And the dynamic biological characteristics of HSPCs senescence may also contribute to the clinical optimal time for antiaging drug intervention.

## 1. Introduction

With abilities of self-renewing, multipotent differentiation, cross-differentiation, and reconstruction of long-term hematopoietic potential, hematopoietic stem cells (HSCs) can differentiate into all hematopoietic lineages throughout the lifetime of an organism. Blood cells are continually produced from the HSCs that reside in the bone marrow (BM). Throughout the lifespan of the organism, the HSC reservoir sustains life. A major property of stem cells is their ability to self-renew, which is important to maintain the HSC pool for the lifespan. Notably, age-related changes in the HSC compartment correlate with organismal aging, which appears as anemia, increased propensity for myeloproliferative neoplasms (MPNs), decreased immune function, and increased cancer incidence [1–4]. Upon aging, the number of HSCs in the BM increases, while their repopulation potential declines. Moreover, aged HSCs exhibit lineage bias in reconstitution experiments with an inclination toward myeloid at the expense of lymphoid potential.

Superoxide damage theory is considered to be one of the key mechanisms of cellular aging; the theory holds that the body has sound oxygen-free radicals generation and scavenging balance system [5]. Since the antioxidant enzymes of body such as superoxide dismutase (SOD) and glutathione (GSH) in the environment of cell survival fall, causing the accumulation of oxygen radicals in cells, cells gradually damage and eventually senescence occur [6]. Although the basic components of the hematopoietic system in aging body are maintained in the process, the quantity and function of HSCs decrease gradually [7].

Stem cell antigen-1 (Sca-1) is the most common marker used to enrich adult murine hematopoietic stem cells (HSCs) and can be used to isolate a nearly pure HSC population when used in conjunction with additional markers [8]. Our recent studies have shown that, compared with Sca-1^+^ hematopoietic stem and progenitor cells (HSPCs) from young mice, those from aged mice showed significantly fewer Sca-1^+^ HSPCs, higher expression of p53, P16^Ink4a^, P19^Arf^, and P21^Cip1^, and decreased telomeric activity. Adhesion ability and CFU-Mix potency were also reduced in Sca-1^+^ HSPCs from aged mice, indicating an aging-related impairment [7, 9, 10].

This study was intended to explore the dynamic biological changes of HSCs aging characteristics. Sca-1^+^ HSPCs were separated and purified from mice at different ages by the magnetic absorptive cell sorting (MACS). We investigated the roles of the superoxide damage and antioxidant capacity in the natural aging process of HSCs, providing experimental evidence and theoretical basis in HSPCs aging mechanism and antiaging drug development.

## 2. Materials and Methods

### 2.1. Ethics Statement and Animal Treatment

The male C57BL/6J mice were purchased from the Medical and Laboratory Animal Center of Chongqing (Animal certificate number: SCXK(yu)2007-0001) and housed in a temperature- and light-controlled room with free access to water and food. All experiments were performed in accordance with institutional and national guidelines and regulations and were approved by the Chongqing Medical University Animal Care and Use Committee. All surgery was performed under sodium pentobarbital anesthesia, and all the efforts were made to minimize suffering.

Animals were divided into 5 groups: Group A (3-4 weeks old, 13–16 g in body weight), Group B (8-9 weeks old, 20–25 g in body weight), Group C (6 months old, 35–40 g in body weight), Group D (12 months old, 39–43 g in body weight), Group E (18 months old, 37–45 g in body weight).

### 2.2. Isolation and Purification of Sca-1^+^ HSPCs in Mouse Bone Marrow

Bone marrow was collected from the femurs and tibias. Mononuclear cells were obtained from BM aspirates by density gradient separation and then subjected to immunomagnetic enrichment of Sca-1^+^ HSPCs (Miltenyi Biotec, Bergisch Gladbach, Germany) as described previously [5–7].

### 2.3. Flow Cytometric Analysis

All flow cytometric analyses were performed using FACSCalibur (BD, Biosciences, Heidelberg, Germany) and data were analyzed with FCS, Express V3 software (De Novo Software, Los Angeles, CA, USA).

### 2.4. Detection of Oxidation-Associated Biomarkers

After the isolation, Sca-1^+^ HSPCs were collected and washed by PBS. GSH content, superoxide content, SOD activity, and MDA content were detected by chemical colorimetric analysis according to the manufacturer's instructions (Beyotime Institute of Biotechnology, Shanghai, China).

### 2.5. Cell-Cycle Analysis of Sca-1^+^ HSPCs

Cell-cycle analysis was performed with the FITC BrdU Flow Kit (BD, Biosciences, Heidelberg, Germany) according to the manufacturer's instructions. Flow cytometric analyses were performed using FACSCalibur (BD, Biosciences, Heidelberg, Germany) and Cell-cycle data were analyzed with Multicycle software (PHENIX, Japan).

### 2.6. The CFU-Mix Formation In Vitro

Initially, 4 × 10^4^ Sca-1^+^ HSPCs were cultivated in 24-well plates (Costar, Corning Incorporated, Corning, NY, USA) with the clonogenic methylcellulose medium H3434 (Stem Cell Technologies, Vancouver, Canada), and cells were cultured in a humidified atmosphere with 5% CO_2_ at 37°C for 2 weeks. Finally, the numbers of CFU-Mix formations were counted under a light microscope.

### 2.7. Senescence-Associated *β*-Galactosidase Cytochemical Staining

The senescence-associated *β*-galactosidase (SA-*β*-gal) activity was determined in Sca-1^+^ HSPCs using the Cellular Senescence Detection Kit (Biolabs, San Diego, CA, USA) according to the manufacturer. In brief, 1 × 10^5^ Sca-1^+^ HSPCs were stained by staining solution for 24 hours at 37°C without CO_2_ in 6-well plates. Cells were counted and the fraction of senescent cells (positive for SA-*β*-gal) was assessed.

### 2.8. RNA Extraction and RT-PCR

6 × 10^6^ Sca-1^+^ HSPCs of each group were collected. Total mRNA was extracted according to the manufacturer's protocol using TRIZOL Reagent (TaKaRA, Japan), OD260/OD280 of RNA: 1.8~2.0. First-strand cDNA was created by AMV reverse transcription Kit (Invitrogen, USA). The unique PCR primers were shown in Table 1. DNA was amplified by an initial incubation at 94°C for 5 min followed by 40 cycles of 94°C denaturation for 15 sec, annealing at 60°C for 60 sec, and 72°C extension for 1 min. PCR product was subjected to 1% agarose gel electrophoresis. Optical intensities were quantified using Quantity One (Bio-Rad). The relative expression levels were calculated as the ratio of the optical intensity of targeted gene to that of the internal reference.

### 2.9. Western Blotting Analysis

To examine the expression of P53, P19, P16^INK4a^, P21^Cip1/Waf1^, cyclinD1, CDK4, CDK2, and CyclinE, which are markers of cell senescence, in freshly isolated Sca-1^+^ HSPCs from each group, we performed western blot analysis. Cells were lysed in buffer [20 mmol/L Tris-HCl, pH 7.5, 150 mmol/L NaCl, 1% Triton X-100, and complete protease inhibitor mixture tablets (Beyotime Institute of Biotechnology, Shanghai, China)] and centrifuged at 1,000 g for 5 min at 4°C. Protein concentrations of the cell were determined by a BCA protein assay reagent kit (Beyotime Institute of Biotechnology, Shanghai, China). The cell lysates (40 *μ*g/well) were subjected to SDS-PAGE and transferred onto a PVDF membrane (Millipore, Bedford, MA, USA). The blotted membranes were incubated with primary antibodies to p53 (Abcam, Cambridge, UK), P19 (Abcam, Cambridge, UK), P16^INK4a^ (ANBO, USA), P21^Cip1/Waf1^ (Ptglab, USA), cyclinD1 (ANBO, USA), CDK4 (Ptglab, USA), CDK2 (ANBO, USA), and CyclinE (Abcam, Cambridge, UK) or ACTB (Ptglab, USA). Corresponding horseradish peroxidase-conjugated antibodies were used as the second antibody (ZSGB-BIO, Beijing, China). The membranes were visualized using the enhanced chemiluminescence detection system (Pierce). The level of *β*-actin was used as an internal control. Relative intensities were quantified using Quantity One (Bio-Rad).

### 2.10. Measurement of Telomere Length by Southern Blot

Telomere lengths were measured from the Sca-1^+^ HSPCs from each group according to the previously described method [11]. In brief, after extraction, DNA was inspected for integrity, digested, resolved by gel electrophoresis, transferred to a membrane, hybridized with labeled probes, and exposed to X-ray film using DAB. The telomere length was measured by Western Biotechnology Corporation.

### 2.11. Detection Activity of Telomerase by Silver Staining TRAP-PCR

The Sca-1^+^ HSPCs were homogenized and lysed on the ice, and the supernatant was collected after centrifugation. The concentrations were measured. The PCR reaction mixture contained 5 *μ*L 10X TRAP buffer, 1 *μ*L dNTPs, 1 *μ*L Taq-DNA polymerase, 1 *μ*L TS primer, and 2 *μ*L extract of telomerase, and 39 *μ*L DEPC water was incubated for 30 min at 23°C for telomerase-mediated extension of the TS primer. The reaction mixture was added 1 *μ*L CX primer to amplify at 94°C for 5 min and then subjected to 35 cycles at 94°C for 30 s, 50°C for 30 s, and 72°C for 90 s. TRAP reaction products were separated by 10% polyacrylamide gel electrophoresis and detected by SYBR green (Gene Inc.) staining. The ratio of enzyme activity = the fluorescence intensity/the protein concentration. Telomerase activity is proportional to the ratio of enzyme activity. The telomerase activity was measured by Western Biotechnology Corporation.

### 2.12. Statistical Analysis

Data were analyzed by *t*-test and ANOVA using SPSS Version 17.0 software and represented as ($\overset{-}{x}\pm s$). Differences were considered significant at *P* < 0.05.

## 3. Results

### 3.1. The Purification of Sca-1^+^ HSC/HPC

**Figure 1:**
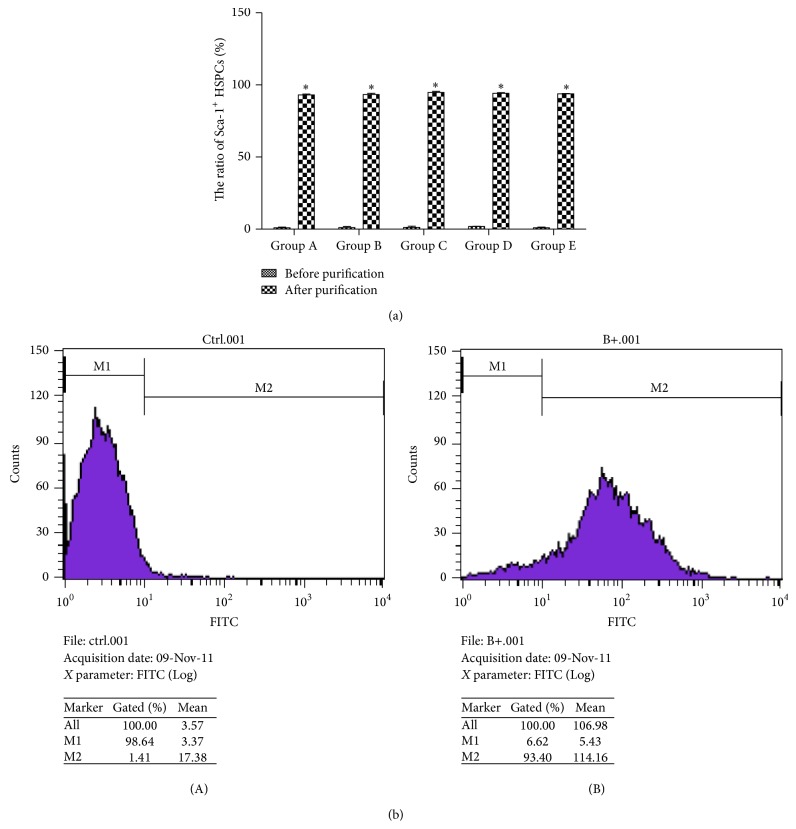
The ratio of sca-1^+^ HSPCs before and after MACS purification. (a) The ratio of sca-1^+^ HSPCs in all groups (%, $\overset{-}{x}\pm s$, *n* = 10). Group A: 3-4-week-old mice, 13–16 g in body weight. Group B: 8-9-week-old mice, 20–25 g in body weight. Group C: 6-month-old mice, 35–40 g in body weight. Group D: 12-month-old mice, 39–43 g in body weight. Group E: 18-month-old mice, 37–45 g in body weight. (b) Result of flow cytometry. (A) The ratio of sca-1^+^ HSPCs before MACS purification. (B) The ratio of sca-1^+^ HSPCs after MACS purification. ^*∗*^ *P* < 0.01, compared with other groups.

Flow cytometry showed that the percentage of Sca-1^+^ HSPCs in collected MNCs was only (1.02 ± 0.19)% before isolation, but the purity of separated Sca-1^+^ HSC/HPS was (93.66 ± 0.83)% after MACS. The differences in five groups were not significant in statistics (Figure 1).

### 3.2. The Superoxide Damage and the Antisuperoxide Ability in Sca-1^+^ HSPCs

**Figure 2:**
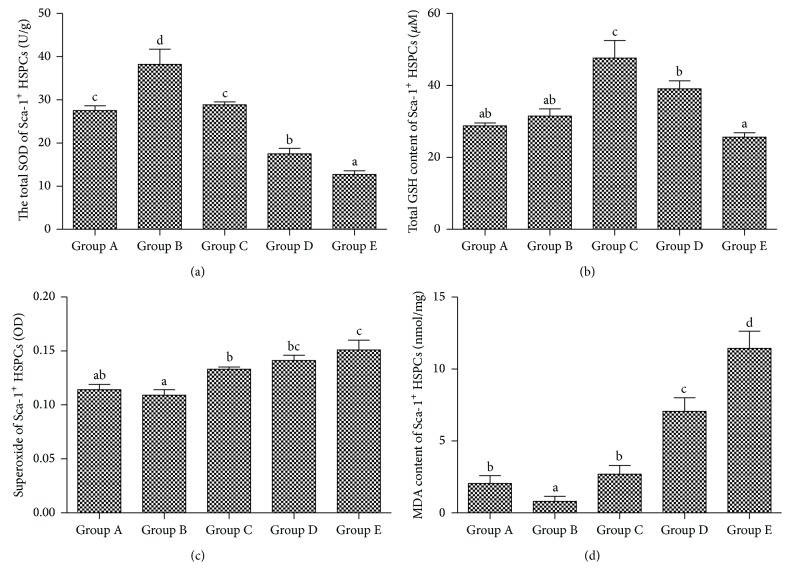
The superoxide damage and the antisuperoxide ability of Sca-1^+^ HSPCs by aging. (a) The activities of SOD of each sample (U/g, $\overset{-}{x}\pm s$, *n* = 10). (b) Glutathione contents in each group (*µ*M, $\overset{-}{x}\pm s$, *n* = 10). (c) The absorbance value of each group which is proportional to superoxide (OD, $\overset{-}{x}\pm s$, *n* = 10). (d) MDA contents of Sca-1^+^ HSPCs in each group (nmol/mg, $\overset{-}{x}\pm s$, *n* = 10). Group A: 3-4-week-old mice, 13–16 g in body weight. Group B: 8-9-week-old mice, 20–25 g in body weight. Group C: 6-month-old mice, 35–40 g in body weight. Group D: 12-month-old mice, 39–43 g in body weight. Group E: 18-month-old mice, 37–45 g in body weight. (a–d) represent a significant difference of these columns (*P* < 0.05).

From group A to E, the activities of SOD of each sample were 27.51 ± 1.11 U/g, 38.23 ± 3.50 U/g, 28.85 ± 0.65 U/g, 17.52 ± 1.27 U/g, and 12.67 ± 0.89 U/g, respectively. It was significantly correlated with changes of age (*r*_2_ = 0.8819, *P* < 0.01) (Figure 2). Glutathione contents in each group were 28.78 ± 0.77 *μ*M, 31.48 ± 2.03 *μ*M, 47.62 ± 4.93 *μ*M, 39.10 ± 2.19 *μ*M, and 25.63 ± 1.23 *μ*M, respectively. A significant correlation was found between GSH contents and ages (*r*_2_ = 0.8754, *P* < 0.01) (Figure 2). From group A to E, the absorbance value of each group which is proportional to superoxide was 0.114 ± 0.005, 0.109 ± 0.005, 0.133 ± 0.002, 0.141 ± 0.005, and 0.151 ± 0.009, respectively. The changes of superoxide levels reflecting absorbance value were found a significant correlation with the ages (*r*_2_ = 0.9464, *P* < 0.05) (Figure 2). MDA contents of Sca-1^+^ HSPCs in each group were 2.06 ± 0.54 nmol/mg, 0.81 ± 0.35 nmol/mg, 2.69 ± 0.62 nmol/mg, 7.06 ± 0.95 nmol/mg, and 11.44 ± 1.19 nmol/mg, respectively. The MDA contents were found to have a positive correlation with the ages (*r*_2_ = 0.9552, *P* < 0.01) (Figure 2).

### 3.3. The Changes of the SA-*β*-Gal Staining in Sca-1^+^ HSPCs by Aging

**Figure 3:**
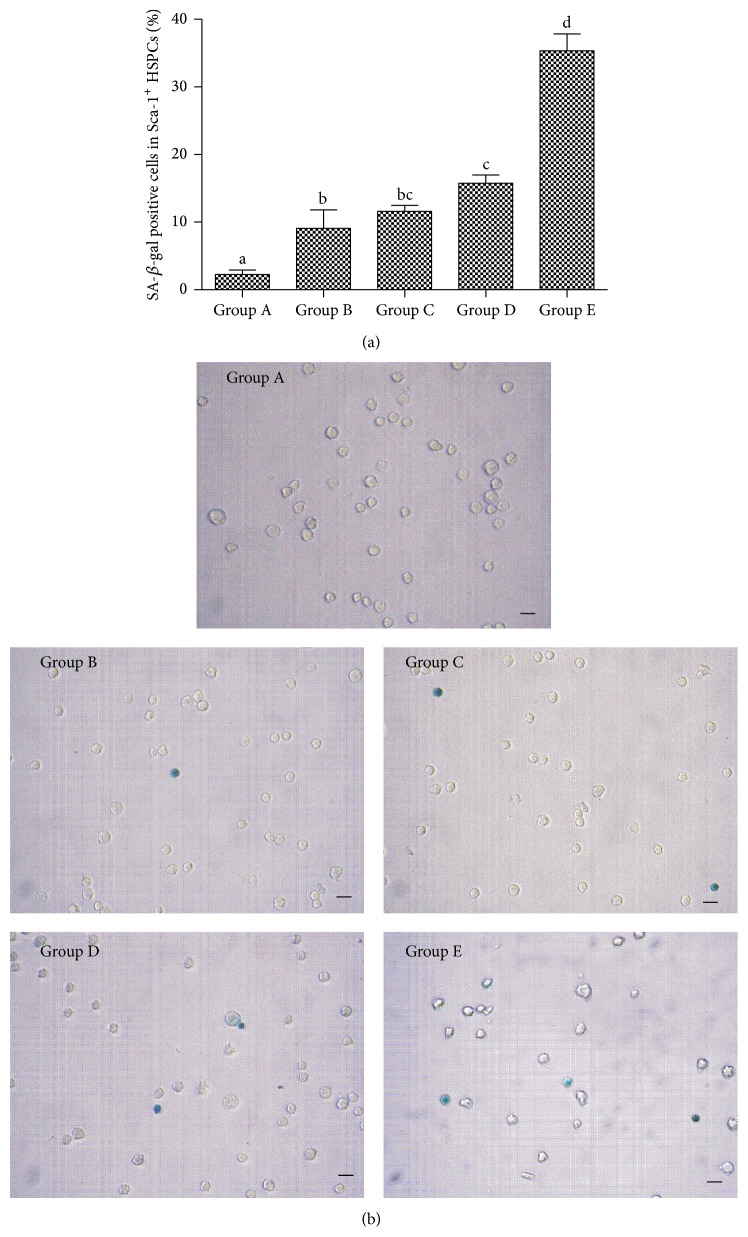
The changes of the SA-*β*-Gal staining in Sca-1^+^ HSPCs by aging. (a) The percentage of SA-*β*-gal positive cells in Sca-1^+^ HSPCs (%, $\overset{-}{x}\pm s$, *n* = 10). Group A: 3-4-week-old mice, 13–16 g in body weight. Group B: 8-9-week-old mice, 20–25 g in body weight. Group C: 6-month-old mice, 35–40 g in body weight. Group D: 12-month-old mice, 39–43 g in body weight. Group E: 18-month-old mice, 37–45 g in body weight. (a–d) represent a significant difference of these columns (*P* < 0.05). (b) Representative micrographs depict morphology of SA-*β*-gal positive cells and SA-*β*-gal negative cells in Sca-1^+^ HSPCs (×400). SA-*β*-gal positive cells are dyed in blue. Scale bars indicate 10 *µ*m.

In SA-*β*-gal staining, aged cells were stained in blue with blue granules in the cytoplasm. SA-*β*-gal positive cells from groups A to E gradually increased significantly (Figure 3).

### 3.4. The Cell Cycle of Sca-1^+^ HSPCs by Aging

**Figure 4:**
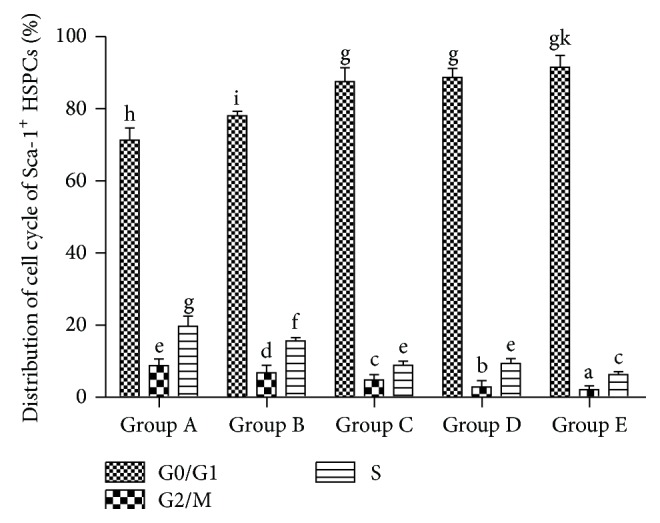
Distribution of cell cycle of Sca-1^+^ HSPCs (%, $\overset{-}{x}\pm s$, *n* = 10). Group A: 3-4-week-old mice, 13–16 g in body weight. Group B: 8-9-week-old mice, 20–25 g in body weight. Group C: 6-month-old mice, 35–40 g in body weight. Group D: 12-month-old mice, 39–43 g in body weight. Group E: 18-month-old mice, 37–45 g in body weight. (a–k) represent a significant difference of these columns (*P* < 0.05).

From Groups A to E, Sca-1^+^ HSPCs of each group displayed significant increased cell-cycle arrests in G1 phase, as the proportion of G0/G1 phase increased, and the proportion of S phase reduced significantly (Figure 4, *P* < 0.05).

### 3.5. The Ratio of CFU-Mix Formation In Vitro of Sca-1^+^ HSPCs by Aging

**Figure 5:**
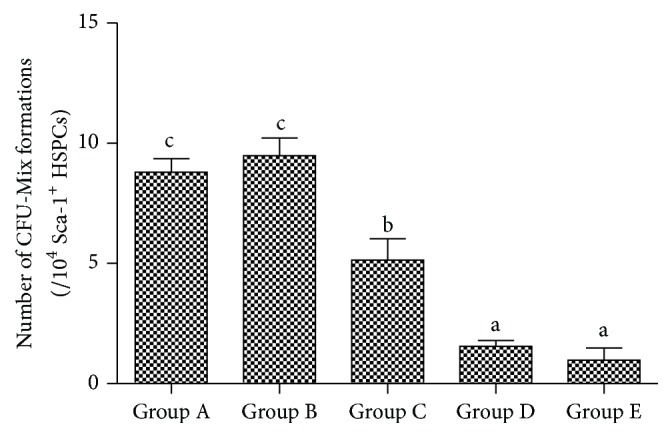
Number of CFU-Mix formations by Sca-1^+^ HSPCs. Group A: 3-4-week-old mice, 13–16 g in body weight. Group B: 8-9-week-old mice, 20–25 g in body weight. Group C: 6-month-old mice, 35–40 g in body weight. Group D: 12-month-old mice, 39–43 g in body weight. Group E: 18-month-old mice, 37–45 g in body weight. (a–c) represent a significant difference of these columns (*P* < 0.05).

The numbers of CFU-Mix formations of Sca-1^+^ HSPCs were reduced gradually from group A to group E, illustrating that the ability of colony formation of Sca-1^+^ HSPCs gradually decreased by aging (Figure 5).

### 3.6. The Effect of Aging on the Expression of Senescence-Associated Genes P21^Cip1^, P16^Ink4a^, P19^Arf^, and P53 mRNA on Sca-1^+^ HSPCs

**Figure 6:**
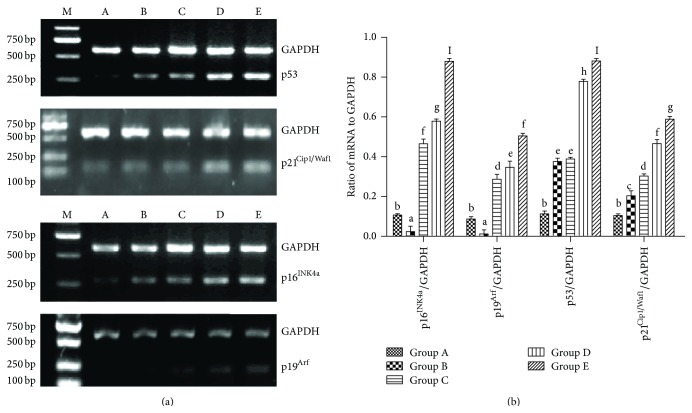
The mRNA expression of p16^INK4a^, p19^Arf^, p53, and p21^Cip1/Waf1^ in Sca-1^+^ HSPCs. (a) Result of RT-PCR. M: DNA mark, A: Group A (3-4-week-old mice, 13–16 g in body weight), B: Group B (8-9-week-old mice, 20–25 g in body weight), C: Group C (6-month-old mice, 35–40 g in body weight), D: Group D (12-month-old mice, 39–43 g in body weight), and E: Group E (18-month-old mice, 37–45 g in body weight). (b) Ratio of mRNA to GAPDH. (a–l) represent a significant difference of these columns (*P* < 0.05).

The results of PCR showed that the expression of p16^INK4a^, p19^Arf^, p53, p21^Cip1/Waf1^, and CyclinD1 mRNA in Sca-1^+^ HSPCs from groups A to E was increased gradually. In group E, the mRNA levels of these genes were significantly higher than the other groups (Figure 6, *P* < 0.05).

### 3.7. The Effect of Aging on the Expression of Senescence-Associated Proteins P53, P19, P16^Ink4a^, P21^Cip1/Waf1^, CyclinD1, CDK4, CDK2, and CyclinE on Sca-1^+^ HSPCs

**Figure 7:**
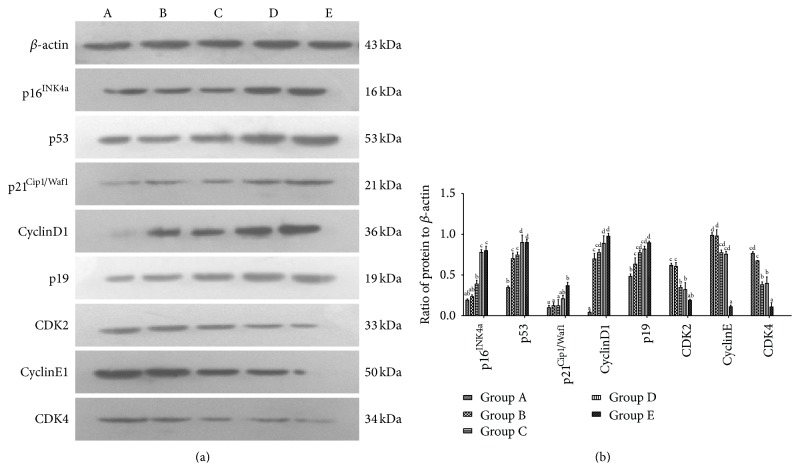
Protein expression of P16^INK4a^, P53, P21^Cip1/Waf1^, CyclinD1, P19, CDK2, CyclinE, and CDK4 in Sca-1^+^ HSPCs. (a) Result of western blot. A: Group A (3-4-week-old mice, 13–16 g in body weight), B: Group B (8-9-week-old mice, 20–25 g in body weight), C: Group C (6-month-old mice, 35–40 g in body weight), D: Group D (12-month-old mice, 39–43 g in body weight), and E: Group E (18-month-old mice, 37–45 g in body weight). (b) Ratio of protein to *β*-actin. (a–d) represent a significant difference of these columns (*P* < 0.01).

By western blotting, the protein levels of P16^INK4a^, P21^Cip1/Waf1^, CyclinD1, P53, and P19 increased gradually, in accordance with the mRNA expression. Meanwhile, the protein levels of CDK4, CDK2, and CyclinE reduced gradually with the aging (Figure 7).

### 3.8. The Effect of Aging on the Telomere Length and Telomerase Activity in Sca-1^+^ HSPCs

**Figure 8:**
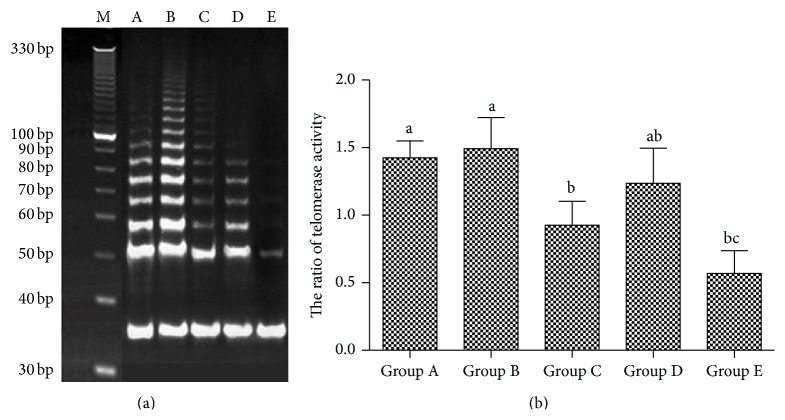
Telomerase activity in the Sca-1^+^ HSPCs. (a) Result of TRAP-PCR. (b) Telomerase activity. M: DNA mark, A: Group A (3-4-week-old mice, 13–16 g in body weight), B: Group B (8-9-week-old mice, 20–25 g in body weight), C: Group C (6-month-old mice, 35–40 g in body weight), D: Group D (12-month-old mice, 39–43 g in body weight), and E: Group E (18-month-old mice, 37–45 g in body weight). (a–c) represent a significant difference of these columns (*P* < 0.01).

**Figure 9:**
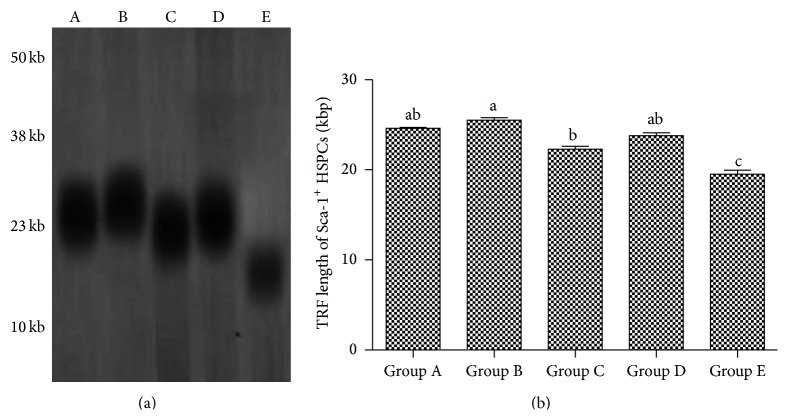
Telomere length of Sca-1^+^ HSPCs. (a) Result of Southern blotting. A: Group A (3-4-week-old mice, 13–16 g in body weight), B: Group B (8-9-week-old mice, 20–25 g in body weight), C: Group C (6-month-old mice, 35–40 g in body weight), D: Group D (12-month-old mice, 39–43 g in body weight), and E: Group E (18-month-old mice, 37–45 g in body weight). (b) Telomere length. (a–c) represent a significant difference of these columns (*P* < 0.05).

From groups A to E, the telomerase activity ratios of Sca-1^+^ HSPCs were 1.424, 1.492, 0.925, 1.235, and 0.570, respectively (Figure 8), while the telomere length was 24.6 kb, 25.5 kb, 22.3 kb, 23.8 kb, and 19.5 kb, respectively (Figure 9).

## 4. Discussion

Theoretically, from the point of view of organs development, 3-4-week-old mice are equivalent to 1-2-year-old human, 2-month-old mice are equivalent to young adults at the age of 15 to 20 years, 6-month-old mice are equivalent to middle-aged human at the age of 40 to 50 years, 12-month-old mice are equivalent to middle-aged human about 60 years old, and 18-month-old mice were equivalent to aged human over 70 years old [12].

As HSCs age, the abilities of self-renewal and differentiation decline. It results in reduced number of HSCs with impaired abilities of proliferation and differentiation. Since the lineages of mature blood cells are differentiated from HSCs, the whole peripheral blood cells decrease, and the risk of aplastic anemia grows. It is shown that HSCs aging and senile leukemia are closely related and, for the elderly people, the risk of acute myeloid leukemia (AML) grows with years [10]. Superoxide dismutase (SOD) is an important antioxidant enzymes in vivo, which can catalyze the disproportionation of superoxide anion and generate hydrogen peroxide (H_2_O_2_) and oxygen (O_2_) [13]. In this study, we found on that SOD contents of Sca-1^+^ HSPCs decreased with age, following a general trend throughout lifespan: from juvenile to young adulthood, it increased rapidly and reached the peak and then gradually decreased. The trend is consistent with the trend of Sca-1^+^ HSPCs development and aging, which indicates that the aging-related decline of antioxidant capacity begins from middle age. Glutathione is an important antioxidant, providing mercapto group to the majority of living cells in vivo. The mercapto group can keep proteins at an appropriate redox state. In the present study, we found that, with increasing age, the overall trend of total glutathione content was similar to the trends of Sca-1^+^ HSPCs formation and development: after reaching the peak at the middle age, it decreased slowly afterwards. It suggests that the aging-related decline of antioxidant capacity begins from middle-age stage.

Superoxide usually refers to superoxide anion O_2_^−^, which is a radical of oxygen molecular and a strong oxidizer. It can be produced by stimulated white blood cells against microbial infection. Superoxide can cause superoxide damage and is closely related with many diseases. In this study, we found that superoxide content increased with aging: from juvenile to young adult, it went down and reached the lowest point which indicated the stronger antioxidant capacity and slowly increased afterwards. This tendency suggests that the superoxide accumulates from the middle age, which is coincident with the Sca-1^+^ HSPCs aging.

MDA is a lipid peroxide produced when radicals attack polyunsaturated fatty acids in the biomembrane. The production of MDA is used as a biomarker to measure the level of superoxide stress in an organism [14]. We found that the contents of MDA in Sca-1^+^ HSPCs stay at low levels for the first two months of the life, maintain stable levels for the next four months, and increase dramatically after the sixth month. This result demonstrated that superoxide damage of Sca-1^+^ HSPCs was slight at the juvenile and young adult stage with the similar levels but increased significantly from the middle-aged stage.

Aging cells usually appear as enlarged size and increase levels of *β*-gal. In this study, we observed that the *β*-gal activity in Sca-1^+^ HSPCs gradually increased, While the colony forming ability decreased by aging, indicating the gradual senescence and function loss of Sca-1^+^ HSPCs.

From the above results, we speculate that, in the process of aging, because of accumulation of risk factors such as the lack of vitamins, metabolic disorders of trace elements, endocrine effects, ultraviolet radiation, or potential threat of ionizing radiation, the antioxidant ability of Sca-1^+^ HSPCs remarkably decreases. It breaks the homeostasis of cellular oxidation and antioxidation, which leads to the increase of superoxide damage. The accumulated superoxide damage may be held responsible for the senescence of Sca-1^+^ HSPCs and the potential risk for senile leukemia and aplastic anemia. Recent study has shown that although the basic components of the hematopoietic system maintain during the aging process, the self-renewal and differentiation potentials of Sca-1^+^ HSPCs decrease, suggesting the senescence of Sca-1^+^ HSPCs [15].

In this study, the cell-cycle analysis by flow cytometry showed that Sca-1^+^ HSPCs displayed G1 phase arrests with aging, with significant increase in proportion of G0/G1 phase and decrease in the proportion of S phase. In this study, RT-PCR and western Blot showed that the mRNA expressions of p16^INK4a^, p19^Arf^, p53, and p21^Cip1/Waf1^ of Sca-1^+^ HSPCs gradually increased with age and protein levels of P53, P19, P16^INK4a^, P21^Cip1/Waf1^, and cyclinD1 gradually increased while CDK4, CDK2, and CyclinE decreased. These changes were consistent with the cell-cycle analysis. This result suggested roles of p16^INK4a^-Rb and p19^Arf^-Mdm2-p53-p21^Cip1/Waf1^ signaling pathways in the natural aging of Sca-1^+^ HSPCs. Telomere length and telomerase activity assay showed the same trend by aging that it increased at first (from the childhood to youth) and then decreased significantly afterwards. Interestingly, telomere length and telomerase activity have a rebound at the elder age but decreased dramatically after that. It suggested that although the senescence of Sca-1^+^ HSPCs starts at middle age, HSCs shows intrinsic antiaging ability to get recovery at the beginning of senescence. However, the antiaging ability was lost gradually when the body got older. Thus, it implied the existence of a spontaneous antiaging ability in the early aging stage. And it also enlightened us that maybe we can improve this antiaging capability by drug intervention or whether the early aging stage is the optimal time for antiaging. These are what we are most interested in and what we want to continue to discuss and research in the future.

## Figures and Tables

**Table 1:** The unique PCR primers.

Primers	Forward (5′-3′)	Reverse (5′-3′)	Length (bp)
p16^INK4a^	TCCGCTGCAGACAGACTGGCCAG	CATCGCGCACATCCAGCCGAGC	295
p19^Arf^	AAGAAGTCTGCGTCGGCGAC	AGTACCGGAGGCATCTTGGACA	215
p53	CACGTACTCTCCTCCCCTCAA	GGCTCATAAGGTACCACCACG	294
p21^Cip1/Waf1^	ATTCCTGGTGATGTCCGACC	AAAGTTCCACCGTTCTCGG	144
GAPDH	GTGCTGAGTATGTCGTGGAGTCT	GAGTGGGAGTTGCTGTTGAAGT	602
